# Combined resection for synchronous lung lesions and esophageal cancer should be compared with staged surgery

**DOI:** 10.1097/JS9.0000000000001309

**Published:** 2024-03-12

**Authors:** Kexun Li, Jie Zhao, Zhenghong Yang, Jie Mao, Yunchao Huang

**Affiliations:** a Department of Thoracic Surgery I, Key Laboratory of Lung Cancer of Yunnan Province, Yunnan Cancer Hospital, The Third Affiliated Hospital of Kunming Medical University, Cancer Center of Yunnan Province; b Department of Thoracic Surgery I, Peking University Cancer Hospital Affiliated Yunnan Hospital, Kunming, People’s Republic of China

*Dear Editor*,

We have read with great interest a paper titled ‘Will synchronous esophageal and lung resection increase the incidence of anastomotic leaks? A multicenter retrospective study’ published in the *International Journal of Surgery* by Liu *et al*.^1^. In this study, the authors conducted a large, multicenter cohort study and utilized propensity score matching (PSM) to adjust for confounding factors. The study not only compared the incidence of anastomotic leaks but also analyzed the trend of survival. The authors suggest that staged surgery might be a more suitable option. While Liu and colleagues have conducted a well-matched and meaningful study, there are certain points that require further discussion.

Firstly, the authors The authors conducted a cohort study comparing the combined resection for synchronous lung lesions and esophageal cancer (CRLE) group with the esophagectomy alone group^1^. However, it is important to note that the study did not compare the CRLE group with the staged surgery group. In our opinion, it is crucial to consider the impact of lung resection, particularly in patients with malignant tumors, on survival outcomes. Additionally, the authors did not specify whether the recurrence pattern observed was related to lung malignancy or esophageal cancer.

Secondly, the significance of preoperative therapy in patients with T2/T3N0-1 esophageal squamous cell carcinoma has been recognized in the CROSS study. This finding was further emphasized in the NEOCRTEC5010 study and is also mentioned in current guidelines^2–5^. However, the study included 258/125 patients with T3 or T4 stages and all patients with more than N1 stages but only 12.5% receive preoperative therapy.

Then, Table 2 provided information on lung resection characteristics in 216 patients. However, only 204 patients (41+61+35+9+58) were included in the analysis of lung lesion location. All 216 patients were included in the analysis of lung lesion type. It is perplexing that they were unable to determine the location of the tumor, but were able to distinguish all the pathological results.

Lastly, numerous studies have consistently found that lymphadenectomy of esophageal cancer has a significant impact on patient survival. This impact is determined not only by the number of lymph nodes but also by the location of the tumor and lymph nodes^6,7^. It is important to note that lung cancer and esophageal cancer have different lymph node reflux modes^8,9^, which means that the lymph nodes that need to be removed may also differ in clinical results^10,11^. These differences could potentially influence survival outcomes.

In summary, although this study holds significant clinical significance, it requires further refinement and discussion of the relevant details to improve the comprehensiveness of the conclusions. It is essential to address these points and delve into the complexities of treatment strategies and surgical considerations in synchronous esophageal and lung lesions. This will be crucial for advancing clinical practice and optimizing outcomes for patients with these challenging conditions.

## Ethical approval

None.

## Sources of funding

This work was supported by the National Natural Science Foundation of China (81960335); National Clinical Key Specialty Construction Project (Thoracic surgery construction project); Science and Technology Department of Yunnan Province (Yunnan Province of Li Yin specialist workstation).

## Author contribution

All authors: study concept and design, acquisition, analysis, or interpretation of data; K.L.: drafting of the article and statistical analysis; Y.H. and J.M.: administrative, technical, or material support and obtained funding; K.L., J.M., and Y.H. had full access to all the data in the study and take responsibility for the integrity of the data and the accuracy of the data analysis.

## Conflicts of interest disclosure

The authors declare that they have no conflicts of interest.

## Research registration unique identifying number (UIN)

None.

## Guarantor

Kexun Li and Yunchao Huang.

## Data availability statement

We are willing to share data, analytic methods, and study materials related to this article with other researchers, provided that all of the above will not be used for commercial or profit purposes. Other researchers can contact the corresponding author of this article by e-mail and indicate the required research materials and purpose. We will gladly provide relevant materials for this study after approval and discussion.

## References

[1] Liu Y Zhou J Gu Y. Will synchronous esophageal and lung resection increase the incidence of anastomotic leaks? A multicenter retrospective study. Int J Surg 2024;110:1653–1662. 38181122 10.1097/JS9.0000000000001018 PMC10942245

[2] Ajani JA D’Amico TA Bentrem DJ. Esophageal and Esophagogastric Junction Cancers, Version 2.2023, NCCN Clinical Practice Guidelines in Oncology. J Natl Compr Canc Netw 2023;4:393–422. 10.6004/jnccn.2023.0019 37015332

[3] Yang H Liu H Chen Y. Neoadjuvant chemoradiotherapy followed by surgery versus surgery alone for locally advanced squamous cell carcinoma of the esophagus (NEOCRTEC5010): a phase III multicenter, randomized, open-label clinical trial. J Clin Oncol 2018;27:2796–2803. 10.1200/JCO.2018.79.1483 PMC6145832 30089078

[4] Wu YY Dai L Yang YB. Long-term survival and recurrence patterns in locally advanced esophageal squamous cell carcinoma patients with pathologic complete response after neoadjuvant chemotherapy followed by surgery. Ann Surg Oncol 2024. doi: 10.1245/s10434-023-14809-1. [Epub ahead of print]. 10.1245/s10434-023-14809-1 38172446

[5] Parente A Kamarajah SK Baia M. Neoadjuvant chemotherapy for intrahepatic, perihilar, and distal cholangiocarcinoma: a national population-based comparative cohort study. J Gastrointest Surg 2023;4:741–749. 10.1007/s11605-023-05606-y PMC10073049 36749556

[6] Li K Leng X He W. Resected lymph nodes and survival of patients with esophageal squamous cell carcinoma: an observational study. Int J Surg 2023;7:2001–2009. 10.1097/JS9.0000000000000436 PMC10389544 37222685

[7] Li K Nie X Li C. Mapping of lymph node metastasis and efficacy index in thoracic esophageal squamous cell carcinoma: a large-scale retrospective analysis. Ann Surg Oncol 2023;9:5856–5865. 10.1245/s10434-023-13655-5 37227576

[8] Rusch VW. Process improvement in lymphadenectomy for lung cancer: the wave of the future? Ann Thorac Surg 2024;117:585. 37717886 10.1016/j.athoracsur.2023.09.007

[9] Xu S He Z Li X. Lymph node metastases in surgically resected solitary ground-glass opacities: a two-center retrospective cohort study and pooled literature analysis. Ann Surg Oncol 2023;6:3760–3768. 10.1245/s10434-023-13235-7 36897416

[10] Uimonen M Helminen O Bohm J. Standard lymphadenectomy for esophageal and lung cancer: variability in the number of examined lymph nodes among pathologists and its survival implication. Ann Surg Oncol 2023;3:1587–1595. 10.1245/s10434-022-12826-0 PMC9908682 36434484

[11] Deng H Zhou J Chen H. Impact of lymphadenectomy extent on immunotherapy efficacy in postresectional recurred non-small cell lung cancer: a multi-institutional retrospective cohort study. Int J Surg 2024;1:238–252. 10.1097/JS9.0000000000000774 PMC10793742 37755384

